# Post-PKS Tailoring Steps of a Disaccharide-Containing Polyene NPP in *Pseudonocardia autotrophica*

**DOI:** 10.1371/journal.pone.0123270

**Published:** 2015-04-07

**Authors:** Hye-Jin Kim, Min-Kyung Kim, Mi-Jin Lee, Hyung-Jin Won, Si-Sun Choi, Eung-Soo Kim

**Affiliations:** Department of Biological Engineering, Inha University, Incheon, Korea; University of Strathclyde, UNITED KINGDOM

## Abstract

A novel polyene compound NPP identified in a rare actinomycetes, *Pseudonocardia autotrophica* KCTC9441, was shown to contain an aglycone identical to nystatin but to harbor a unique di-sugar moiety, mycosaminyl-(α1-4)-*N*-acetyl-glucosamine, which led to higher solubility and reduced hemolytic activity. Although the *nppDI* was proved to be responsible for the transfer of first polyene sugar, mycosamine in NPP biosynthesis, the gene responsible for the second sugar extending glycosyltransferase (GT) as well as NPP post-PKS tailoring mechanism remained unknown. Here, we identified a NPP-specific second sugar extending GT gene named *nppY*, located at the edge of the NPP biosynthetic gene cluster. Targeted *nppY* gene deletion and its complementation proved that *nppY* is indeed responsible for the transfer of second sugar, *N*-acetyl-glucosamine in NPP biosynthesis. Site-directed mutagenesis on *nppY* also revealed several amino acid residues critical for NppY GT function. Moreover, a combination of deletions and complementations of two GT genes (*nppDI* and *nppY*) and one P450 hydroxylase gene (*nppL*) involved in the NPP post-PKS biosynthesis revealed that NPP aglycone is sequentially modified by the two different GTs encoded by *nppDI* and *nppY*, respectively, followed by the *nppL*-driven regio-specific hydroxylation at the NPP C10 position. These results set the stage for the biotechnological application of sugar diversification for the biosynthesis of novel polyene compounds in actinomycetes.

## Introduction

Invasive fungal infections are becoming a serious problem in human health. The mortality rates for fungal infections with three most common species of pathogens are *Candida albicans* of 20%-40%, *Aspergillus fumigatus* of 50%-90%, and *Cryptococcus neoformans* of 20%-70% [1–3]. These diseases usually occur in the setting of immunocompromised patients as a result of HIV infection, anticancer therapy, or immunosuppressive therapy after organ transplantation [1]. Azoles and polyenes have been classified as two major classes of antifungal drugs [4–6]. Although azoles inhibit the fungal cytochrome P450 (CYP)-dependent enzyme involved in ergosterol biosynthesis, the emergence of *Candida* resistance to azoles has become an increasing concern [7]. Meanwhile, polyene macrolides including nystatin and amphotericin exhibit their antifungal activities through direct binding to fungal membrane ergosterol [8, 9]. Although broad-spectrum fungicidal activity and low incidence of fungal resistance were considered as main benefits of polyenes, high toxicity and low water solubility limit their broad clinical application [10, 11]. In recent years, genetic engineering has become a promising strategy for design of new analogues of polyene with improved pharmacological properties [9, 12–15].

Polyene macrolides are usually biosynthesized by large modular type I polyketide synthases (PKSs), followed by several sequential post-PKS modifications. During post-PKS modification, a unique cytochrome P450 hydroxylase (CYP) regio-specifically transfers a hydroxyl group to a specific carbon position of the polyene backbone, and a polyene glycosyltransferase (GT) catalyzes addition of mycosamine, a deoxyaminosugar derived from GDP-d-mannose. GTs are an important class of enzymes and are essential for the biosynthesis of glycosylated natural products [16–18], and this glycosylation reaction often converts a non-bioactive intermediate into a biologically active compound [19]. Although most polyene compounds contain a single sugar mycosamine attached, a few polyenes naturally contain a disaccharide with an additional sugar residue to mycosamine. 67-121C, a disaccharide-modified aromatic heptaene, has been isolated from *Actinoplanes caeruleus* [20]. Another example, nystatin P1 containing a disaccharide mycosamine-glucose was also identified in the *Pseudonocardia* P1 strain collected from *Acromyrmex octospinosus* garden worker ants [21]. Although the genes for the second sugar extending GTs in 67-121C and nystatin P1 were proposed as *pegA* and *nypY*, respectively [20, 21], their functional confirmation and post-PKS mechanisms have been poorly understood.

**Fig 1:**
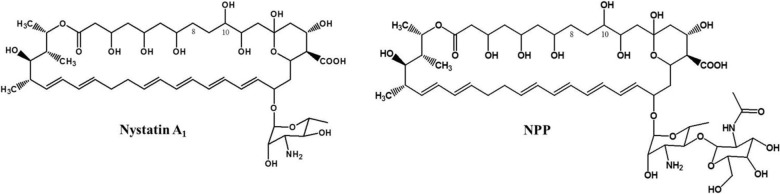
Chemical structures of nystatin A1 and NPP. These structurally related compounds share the same aglycone with mono-sugar, mycosamine. NPP include additional sugar moiety, N-acetylglucosamine.

Previously, a rare actinomycetes called *Pseudonocardia autotrophica* was determined to contain a cryptic polyene biosynthetic gene cluster through a polyene CYP-specific genome screening strategy [22], and then a novel nystatin-like polyene (NPP) containing a disaccharide, mycosamine (α 1–4)-N-acetyl-2-aminoglucose was identified (Fig 1) [23]. Interestingly, NPP harboring a disaccharide moiety showed significantly higher solubility and lower hemolytic activity than nystatin which contains a single sugar mycosamine only, suggesting that NPP could be a promising candidate for further development into a pharmacokinetically-improved, less-cytotoxic polyene antifungal antibiotic. The first GT involved in NPP biosynthetic pathway, NppDI was confirmed to be homologous to a previously-identified NysDI, which is responsible for the transfer of the first sugar, mycosamine to the C19 position of nystatin in *S*. *noursei* ATCC11455. Although the additional sugar, *N*-acetyl-glucosamine present in NPP is believed to be the major reason for the increased solubility and the reduced toxicity [23], the gene for this NPP-specific second sugar extending GT was not found in the previously-reported biosynthetic gene cluster. In this report, we identified a NPP-specific second sugar extending GT through *P*. *autotrophica* genome mining, targeted gene disruption, and complementation. In addition, NPP post-PKS biosynthetic modification steps including regio-specific hydroxylation and two glycosylations were completely elucidated in *P*. *autotrophica*.

## Materials and Methods

### Bacterial Cultures

The bacterial strains and plasmids used in the present study are described (Table 1). The rare actinomycetes *Pseudonocardia autotrophica* KCTC 9441 was purchased from the Korean Collection for Type Cultures. The *P*. *autotrophica* strain was maintained on ISP2 agar medium containing (grams per liter) 4g glucose, 4g yeast extract, and 10g malt extract at 28°C for the preparation of spore [22], and was cultured in YEME liquid medium composed of 3g yeast extract, 5g peptone, 3g malt extract, 10g glucose, 340g sucrose, 2mL MgCl_2_ 6H_2_O (2.5M) in 1L of distilled water for NPP production [23]. Cultivations of S. noursei strains were performed as described previously [24]. All *Escherichia coli* strains were incubated at 37°C in Luria-Bertani broth or on Luria-Bertani agar supplemented with the appropriate antibiotics when needed [25].

**Table 1:** Bacterial strains and plasmids used in this study.

Strain of plasmid	Characteristic^a^	Source or reference
**Strain**		
*Escherichia coli*		
DH5α	General cloning host	
ET12567 / pUZ8002	Strain for intergeneric conjugation; Km^r^, Cm^r^	[37]
*Pseudonocardia autotrophica*		
KCTC9441	Wild-type, NPP producing strain	KCTC
ESK601	*nppDI* mutant producing 10-deoxyaglycone	[23]
ESK602	*nppDI*-complemented ESK601	[23]
ESK6011	*nppY* mutant producing 10-deoxynystatin	This work
ESK6012	*nppY*-complemented ESK6011	This work
ESK60111	ESK6011 harboring plasmid pYM1	This work
ESK60112	ESK6011harboring plasmid pYM2	This work
ESK60113	ESK6011 harboring plasmid pYM3	This work
ESK60114	ESK6011 harboring plasmid pYM4	This work
ESK60115	ESK6011 harboring plasmid pYM5	This work
ESK60116	ESK6011 harboring plasmid pYM6	This work
ESK60117	ESK6011 harboring plasmid pYM7	This work
ESK60118	ESK6011 harboring plasmid pYM8	This work
ESK60119	ESK6011 harboring plasmid pYM9	This work
ESK6021	*nppL* mutant producing C10 deoxy NPP	This work
ESK6022	*nppL*-complemented ESK6021	This work
ESK611	*nppDI nppY* double mutant producing 10-deoxyaglycone	This work
ESK612	*nppDI*-complemented ESK611	This work
ESK613	*nppY*-complemented ESK611	This work
ESK614	*nppDI nppY*-double complemented ESK611	This work
ESK621	*nppDI nppL* double mutant producing 10-deoxyaglycone	This work
ESK622	*nppDI*-complemented ESK621	This work
ESK623	*nppL*-complemented ESK621	This work
ESK624	*nppDI nppY*-double complemented ESK621	This work
ESK631	*nppY nppL* double mutant producing 10-deoxynystatin	This work
ESK632	*nppY*-complemented ESK631	This work
ESK633	*nppL*-complemented ESK631	This work
ESK634	*nppY nppL*-double complemented ESK631	This work
ESK641	*nppDI nppY nppL* triple mutant producing 10-deoxyaglycone	This work
ESK642	*nppDI*-complemented ESK641	This work
ESK643	*nppDI nppY*-complemented ESK641	This work
ESK644	*nppDI nppY nppL*-complemented ESK641	This work
**Plasmid**		
pTY	Template plasmid for *nppY* mutation, Am^r^	This work
pDELY	Plasmid for deletion *nppY* gene, Apr^r^	This work
pDELL	Plasmid for deletion *nppL* gene, Apr^r^	This work
ermE*pSET152	*E*. *coli-P*. *autotrophica* conjagative vector with constitutive promoter, ermE*, Apr^r^	[38]
pMJPDI	Plasmid for *nppDI* complementation, Apr^r^	[23]
pPY	Plasmid for *nppY* complementation, Apr^r^	This work
pPL	Plasmid for *nppL* complementation, Apr^r^	This work
pPDIY	Plasmid for *nppDI nppY* complementation, Apr^r^	This work
pPDIL	Plasmid for *nppDI nppL* complementation, Apr^r^	This work
pPYL	Plasmid for *nppY nppL* complementation, Apr^r^	This work
pYM1	pPY with additional 7 amino acids (FARSYTP), Hyg^r^	This work
pYM2	pPY with A37N mutation, Hyg^r^	This work
pYM3	pPY with E197K mutation, Hyg^r^	This work
pYM4	pPY with R200N mutation, Hyg^r^	This work
pYM5	pPY with I201Q mutation, Hyg^r^	This work
pYM6	pPY with R219V mutation, Hyg^r^	This work
pYM7	pPY with R247E mutation, Hyg^r^	This work
pYM8	pPY with I385Y mutation, Hyg^r^	This work
pYM9	pPY combined with C-terminal domain of NppDI, Hyg^r^	This work

^a^Km^r^, kanamycin resistance; Cm^r^, chloramphenicol resistance; Am^r^, ampicillin resistance; Apr^r^, apramycin resistance; Hyg^r^, hygromycin resistance.

### Production and Purification of NPP

The large-scale fermentation was performed in YEME medium (3L). NPP producing strain, *P*. *autotrophica* spores were inoculated to ISP medium 2 (6 x 50mL) incubated (28°C; 220 rpm) for 72hr. All precultures were added to YEME autoclaved at 121°C for 20 min and 150g Amberlite XAD16 resin was supplied after 48hr cultivation. After 24hr, the mycelia and resin from production media were isolated and extracted two times with 600ml butanol. The organic phase was concentrated using a vacuum evaporator. The raw extract was dissolved in methanol and loaded onto column packed with C18 reversed-phase silica gel (Daiso) with methanol-water (30:70, v/v) to remove sugar originating from production media. The fractions were separated and purified using fraction collector (Interchim, France) with solvent A (water) and solvent B (methanol) following gradient: 30% B (v/v) (0 to 10 min), 100% B (v/v) (100 min) at a flow rate of 15ml/min. The fractions containing NPP with >90% purity were judged by HPLC analysis with detection at 305nm.

### HPLC analysis of polyene analogues

Polyene macrolides were extracted with an equal volume of butanol, followed by concentration and dissolving in methanol. HPLC analysis of these extract were carried out using a Shimadzu SPD M10A (Shimadzu, Japan) with a Zorbax SB-C_18_ column (5 μm particles, 4.6 x150 mm, Agilent). The column was equilibrated with 50% solvent A (0.05 M ammonium acetate, pH 6.5) and 50% solvent B (methanol), followed by development using the following gradient: 50% B (0 min), 75% B (3 min), 100% B (30 min), 50% B (33 min), and 50% B (40 min) at a flow rate of 1.0 ml/min and UV/Vis detection at 305 nm [26]. NPP quantification was performed with HPLC using the authentic Nystatin A1 (Sigma Aldrich) as a standard, of which the method was also described elsewhere [12, 22, 23]

### Targeted gene deletion

**Fig 2:**
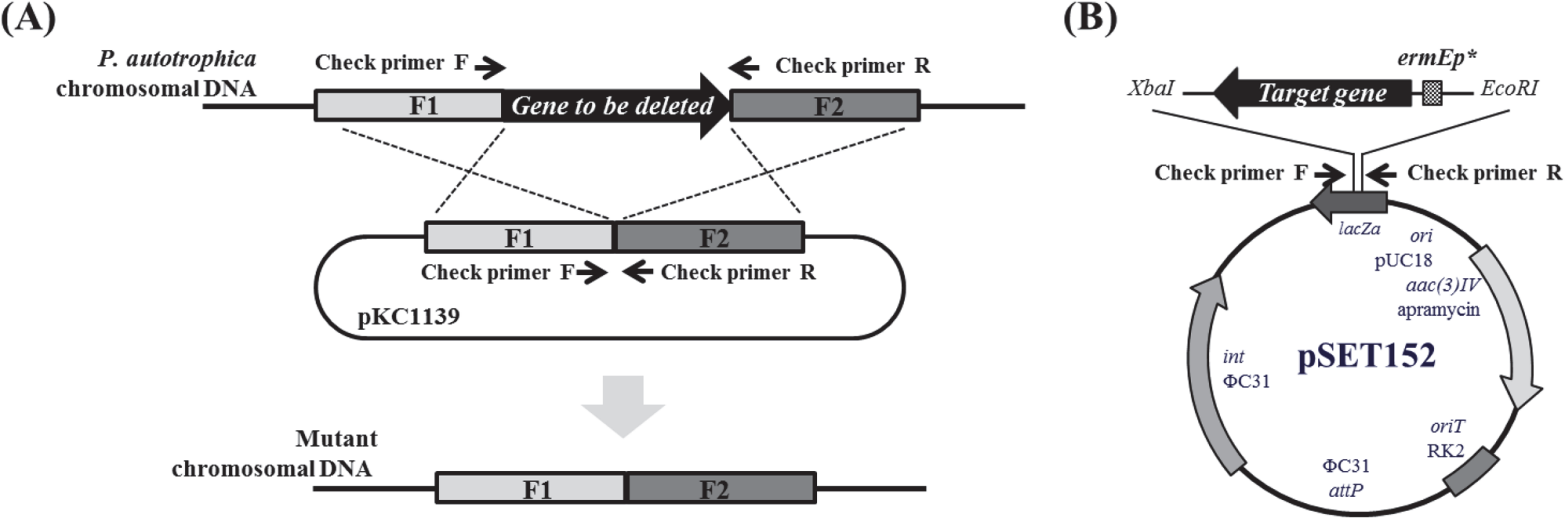
Genetic manipulations for targeted gene disruption and complementation. (A) A scheme representation of the in-frame deletion mutant by double homologous recombination. (B) Plasmid map of the *Streptomyces* integrative vector, pSET152 for expression of target genes under the control of strong constitutive promoter, ermE*.

*nppY*-deletion mutant, ESK6011 and *nppL*-deletion mutant, ESK6021 were constructed by double homologous recombination (Fig 2A). To construct both double knock-out mutants (ESK611, ESK621, ESK631) in *P*. *autotrophica*, an identical strategy for genetically targeted gene deletion was applied using bi-functional *E*. *coli*-*Streptomyces* and temperature-sensitive plasmid, pKC1139. This plasmid contained two 1.5 kb fragments homologous to upstream and downstream of targeted gene to be deleted. Each of the fragments was individually amplified using the polymerase chain reaction (PCR) (BioRad, USA). All PCR primer sequences used in this work are listed (S1 Table). Amplified fragments were sequenced and ligated into pKC1139 digested with *Hind*III-*EcoR*I. The constructed plasmid was introduced into *P*. *autotrophica* using conjugation. The spores containing pKC1139 derivative were selected and sub-cultured three times in broth media with apramycin at 37°C. The collected spores were cultivated in broth media without apramycin at 30°C After three repeats of sub-culture, several double cross-over apramycin-sensitive recombinants were identified *via* PCR amplification of the deleted-junction.

### Functional expression and complementation

For complementation of gene deleted mutant and functional gene expression, the integrative plasmid pSET152 harboring the strong constitutive promoter, ermE* was utilized (Fig 2B). Oligonucleotide sequences and templates applied for amplifying targeted gene using PCR were shown (S1 Table). Amplified genes were cloned into pSET152 using In-Fusion HD Cloning Kit (Clontech, Japan) and were sequenced. The resulting plasmids were introduced into *P*. *autotrophica via* intergeneric conjugation and apramycin was used for selection [27].

### Mutagenesis of NPP-specific glycosyltransferase (NppY) and cytochrome P450 (NppL)

pTY, in which the *nppY* gene had been subcloned into T&A cloning vector (RBC) was used as the template for site-directed mutagenesis and construction of the hybrid fusion protein. The site-directed mutations were introduced by using the Q5 Site-Directed Mutagenesis Kit (New England BioLabs Inc.) and hybrid fusion proteins were constructed using In-Fusion HD Cloning Kit (Clontech, Japan). All PCR primer pairs are listed in S1 Table. About 1.5kb fragment emcompassing the modified *nppY* coding sequences were excised with *BamHI*/*XbaI*, and ligated with the integrative plasmid pSET152 containing ermE* promoter. The resulting recombinant plasmids were introduced into the ESK6011 strain. The DNA sequence of *nppY* was deposited in GenBank under the accession number KP419743.

## Results

### Identification of a NPP-specific extending GT

**Fig 3:**
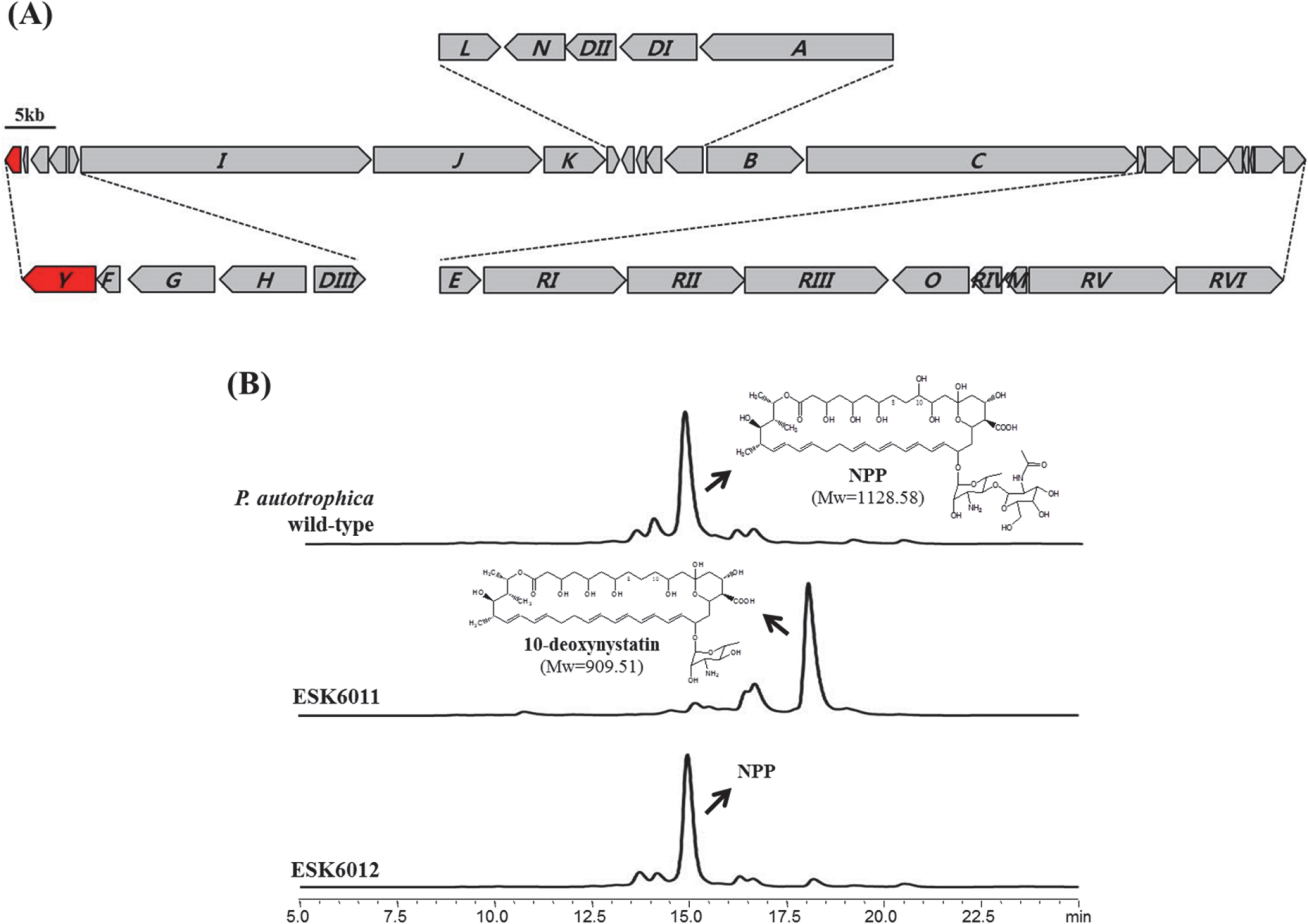
NPP biosynthetic gene cluster and disruption-complementation of *nppY*. (A) Organization of the NPP biosynthetic gene cluster. The *nppY* gene (red highlight) is located immediately upstream of the *nppF* gene. NppF, phosphopantetheinyl transferase; NppG and NppH, ABC transporter; NppDIII, GTP-mannose-4,6-dehydratase; NppI, NppJ, NppK, NppA, NppB, and NppC, Type I PKS; NppL and NppN, P450 mono oxygenase; NppDII, aminotransferase; NppDI, glycosyltransferase; NppE, thioesterase; NppRI, NppRII, NppRIII, NppRIV, NppRV, and NppRVI, regulatory enzyme; NppO, decarboxylase; NppM, ferredoxin. (B) HPLC profiles and chemical structures of culture extracts from *P*. *autotrophica* wild-type, ESK6011, and ESK6012.

To isolate a NPP-specific extending GT responsible for the second sugar transfer, the *P*. *autotrophica* KCTC9441 genome was sequenced by GenoTech, Korea. The draft genome sequence of *P*. *autotrophica* comprised 9,977,725 bases, assembled into 1,016 contigs (>500bp), and it had a GC content of 69.9%. Further, there were 96 predicted tRNAs sequences along with 10,581 protein-coding sequences (CDSs) in the genome sequence. Specifically, the coding percentage was 70.5%, and 7,466 CDSs showed functional predictions. Using COG (Clusters of Orthologous Group) functional assignment, the majority of predicted proteins were classified into 25 COG categories. Using *in silico* analysis of amino acid sequences performed by GenoTech Prokaryotic Genome Automatic Annotation System, a total of 112 putative glycosyltransferase (GT) gene was identified from *P*. *autotrophica* whole genome (S2 Table). Among *P*. *autorhophica* GT genes, one GT gene located in the next *nppF* coding phosphopantetheinyl transferase in NPP biosynthesis, was identified (Fig 3A). The translational product of that gene exhibited 42% identity to NppDI which is responsible for catalyzing the transfer of first sugar, mycosamine during NPP biosynthesis (S3 Table). Interestingly, this gene product also displayed 83% amino acid identity to NypY, which adds a hexose to the mycosamine of a nystatin in *Pseudonocardia* sp. P1 strain [28]. Since the *nypY*, present in the similar location in the nystatin P1 cluster, was recently proposed to encode a GT responsible for second sugar transfer [29], this gene was accordingly named as *nppY* encoding a putative NPP-specific extending GT. NppY also showed 51% identity to PegA which is the GT that transfers the second sugar to biosynthesize 67-121C polyene macrolide in *Couchioplanes caeruleus*.

### Deletion and Complementation of NPP-specific second sugar extending GT

To verify the function of putative NPP-specific second sugar extending GT, deletion of the *nppY* was performed using a temperature-sensitive suicide plasmid, pKC1139 (Fig 2A). Construction of the *nppY*-deleted mutant, ESK6011 was confirmed by PCR analysis (S1A Fig) and HPLC-MS analysis (Fig 3B). Since the additional sugar present in NPP is the only difference between NPP and nystatin, ESK6011 was predicted to produce nystatin with the expected mass of 926.09. Interestingly, however, the polyene compound produced in ESK6011 was a 10-deoxynystatin with the mass of 909.51, indicating that the polyene analogue accumulated in ESK6011 lacked both the second sugar moiety and the C10 hydroxyl group. Through co-injection experiment with the 10-deoxynystatin produced by the *nysL* inactivation mutant [29], we could confirm that the polyene derivative resulted from the *nppY* deletion mutant is indeed 10-deoxynystatin (S1B Fig). When *nppY* gene was functionally expressed in a pSET152 vector under the ermE* promoter in the ESK6011 (named ESK6012), the NPP production was restored (Fig 3B and S1B Fig). Considering the fact that the functional NppL responsible for C10 hydroxylation is still present in the ESK6011, these results suggest that the NPP C10 hydroxylation might be catalyzed after the completion of NppY-driven second glycosylation (see more below).

### Molecular Dissection of NPP-specific Extending GT

**Fig 4:**
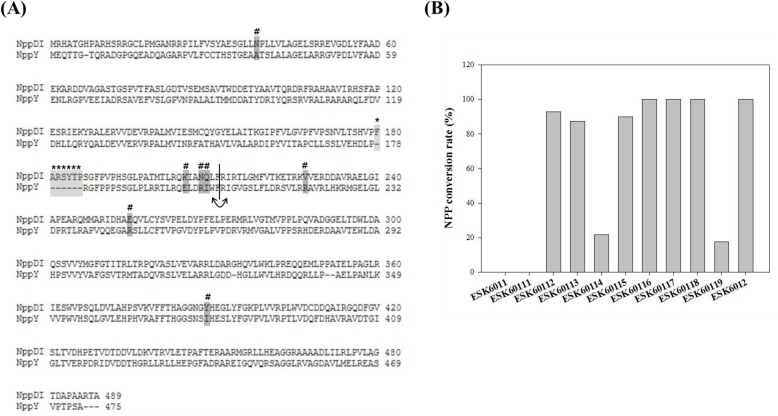
Amino acid sequence alignment and mutagenesis of NppY. (A) Amino acid sequence alignment between NppDI and NppY glycosyltransferases in NPP biosynthesis. Regions for mutagenesis are marked; crosshatch, site-directed mutagenesis (dark gray); asterisk, addition of 7 consecutive amino acids (FARSYTP) from NppDI (light gray); arrow, N-terminal of NppY and C-terminal of NppDI were combined based on straight line. (B) NPP conversion rate through expression of various modified *nppY* genes in ESK6011.

To increase understanding of the NPP-specific second sugar extending GT, further investigation of the key amino acid residues of NppY was pursued. We analyzed the amino acid sequence of NppY in specialized BLAST based on the TDP-vancosaminyltransferase GtfD involved in the final stage of vancomycin biosynthesis (S2 Fig) [30]. Through comparing amino acids with GtfD, we deduced putative active sites, acceptor binding sites, and activated NDP-sugar donor binding sites of the NppY protein. Alignment of the amino acid between NppY and NppDI would also be the clue to find donor interacting domain, because these proteins could dedicate the transfer of different sugar moiety for NPP biosynthesis in *P*. *autotrophica* (Fig 4A). To verify which amino acid residues are essential for function of NppY, we substituted some amino acid residues of NppY with those of NppDI at the predicted critical position. In addition, consecutive amino acids (FARSYTP) present only in the NppDI were inserted into the corresponding position in NppY. A fusion protein between NppY and NppDI was also generated. HPLC analyses showed that NPP productions in ESK60116 (R219V), ESK60117 (R247E), and ESK60118 (I385Y) had no significant change compared to the control, which is the ESK6011 complemented with the intact *nppY* gene (ESK6012). ESK60112 (A37N), ESK60113 (E197K), and ESK60115 (I201Q) led to reductions of the NPP production by approximately 20%. More than 80% decreases of NPP production in ESK60114 (R200N) and ESK60119 (fusion between N-terminal of NppY and C-terminal of NppDI) were observed. Moreover, addition of seven amino acid residues (FARSYTP) present only in NppDI into the corresponding sites of NppY (ESK60111) led to a complete loss of NppY enzyme activity (Fig 4B).

### Post-PKS Tailoring Steps of NPP

Although three genes including two GT genes (*nppDI* and *nppY*) and one CYP gene (*nppL*) are now proposed to be required in NPP post-PKS biosynthesis, the NPP post-PKS tailoring cascade including the order of these three steps is not clearly understood. Based on the results of *nppY* disruption-&-complementation, one could hypothesize that the NppL might not be able to hydroxylate the deoxynystatin at the C10 position as a substrate, despite of the high degree sequence homology between NysL and NppL (68% identity). To elucidate the NPP post-PKS tailoring steps, single knockout mutants of, ESK601(*ΔnppDI*), ESK6011(*ΔnppY*), and ESK6021(*ΔnppL*) as well as double knockout mutants ESK611(*ΔnppDI ΔnppY*), ESK621(*ΔnppDIΔnppL*), and ESK631(*ΔnppYΔnppL*) were generated (Table 2). Subsequently, each mutant was complemented by the expression construct containing either single (*nppDI*, *nppY*, and *nppL*) or double (*nppDI/nppY* and *nppDI/nppL*) genes (Table 2).

**Table 2:** Generated *P*. *autotrophica* mutant strains and compounds through both gene deletion and complementation.

Deletion		Complementation	
Mutant strain	Resulted compound	Complementing gene	Resulted compound
ESK601(Δ*nppDI*)	10-deoxyaglycone	*nppDI*(ESK602)	NPP
ESK6011(Δ*nppY*)	10-deoxynystatin	*nppY*(ESK6012)	NPP
ESK6021(Δ*nppL*)	10-deoxyNPP	*nppL*(ESK6022)	NPP
ESK611(Δ*nppDI*Δ*nppY*)	10-deoxyaglycone	*nppDI*(ESK612)	10-deoxynystatin
		*nppY*(ESK613)	10-deoxyaglycone
		*nppDI&nppY*(ESK614)	NPP
ESK621(Δ*nppDI*Δ*nppL*)	10-deoxyaglycone	*nppDI*(ESK622)	10-deoxyNPP
		*nppL*(ESK623)	10-deoxyaglycone
		*nppDI&nppL*(ESK624)	NPP
ESK631(Δ*nppY*Δ*nppL*)	10-deoxynystatin	*nppY*(ESK632)	10-deoxyNPP
		*nppL*(ESK633)	10-deoxynystatin
		*nppY&nppL*(ESK634)	NPP
ESK641(Δ*nppDI*Δ*nppY*Δ*nppL*)	10-deoxyaglycone	*nppDI*(ESK642)	10-deoxynystatin
		*nppDI&nppY*(ESK643)	10-deoxyNPP
		*nppDI&nppY&nppL*(ESK644)	NPP

**Fig 5:**
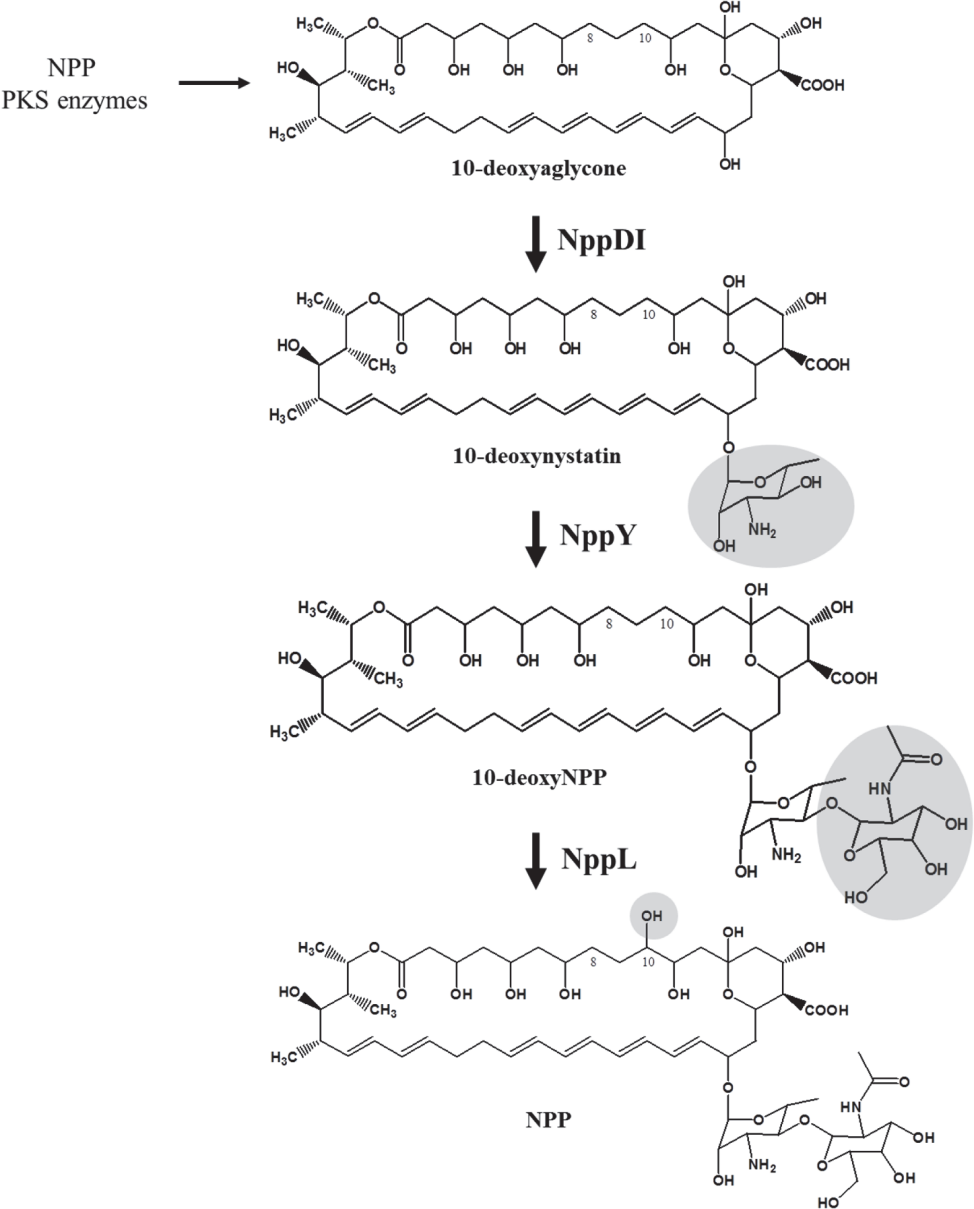
A proposed post-PKS modification pathway in the NPP biosynthesis.

The *nppL* gene in the *P*. *autotrophica* chromosome is located downstream of *nppK* encoding the last module of the NPP PKS. Since the experimental evidence for NysL which leads to nystatin production from 10-deoxynystatin was suggested [29], we could predict the role of NppL in production of NPP. The *nppL* gene was inactivated via double homologous recombination in *P*. *autotrophica* chromosomal DNA. The polyene compound produced in ESK6021 was confirmed to be 10-deoxyNPP using HPLC-MS analysis (S3 Fig). The functional expression of *nppL* gene under the control of the constitutive promoter, ermE* in the ESK6021 restored NPP production, suggesting that NppL-driven hydroxylation should be the last step converting 10-deoxyNPP to NPP (S3 Fig). When the *nppY* gene was functionally expressed under the ermE* promoter in the double knock-out mutant ESK631, the 10-deoxyNPP was produced (S4 Fig). In addition, the expression of the *nppDI* gene in the double knock-out mutant ESK621 also generated 10-deoxyNPP. All these results also indicate that NppY catalyzes the transfer of second sugar, N-acetylglucosamine first, and then the NppL-driven regio-specific hydroxylation is the final post-PKS modification step in NPP biosynthesis (Fig 5). The proposed post-PKS tailoring steps of NPP biosynthesis was re-confirmed by a series of complementation in a triple knockout mutant (*ΔnppDIΔnppYΔnppL*) (Table 2 and S4 Fig).

## Discussion

Polyene macrolides such as amphotericin B and nystatin are very effective broad spectrum antifungal antibiotic that is active against human immunodeficiency virus and prion diseases. Unfortunately, the therapeutic application of polyene macrolides is restricted by severe side effects that can be moderated by structural alteration or liposomal formulation [31, 32]. Using the genetic information of various polyene biosynthetic gene clusters, a number of approaches have been used to address the problems associated with their medical use, such as generation of both semisynthetic and genetically-engineered polyene analogues. Despite several promising results, no new polyene-based antifungal agent has been applied in the clinic, except for in lipid and liposomal formulations [33], suggesting that the search for novel polyene macrolide antifungals for human use remains a challenging task. Recently, a rare actinomycetes, *P*. *autotrophica* KCTC9441, was shown to contain genes likely to encode compounds involved in polyene biosynthesis and were named NPP. These structural studies revealed that the aglycone of NPP is identical to the aglycone of nystatinolide. However, we have shown that NPP includes a unique disaccharide moiety, mycosamine (α1–4)-*N*-acetyl-glucosamine, linked at the C-19 hydroxyl position. In order to examine the unusual sugar moiety, *nppDI* was investigated using gene inactivation and complementation with the *nppDI* and *nysDI* genes. Surprisingly, *nppDI* could be substituted by *nysDI*, which can be explained by the existence of an alternative glycosyltransferase (GT) capable of attaching *N*-acetyl-glucosamine to nystatin. The alternative GT gene could be crucial for further investigation of the other polyenes with disaccharide moieties.

In this present study, we demonstrated that NPP-specific second sugar extending GT, NppY was responsible for the transfer of the second sugar, N-acetylglucosamine to 10-deoxynystatin through *P*. *autotrophica* genome mining, targeted-gene inactivation and complementation. The genetic deletion of *nppY* in *P*. *autotrophica* chromosomal DNA completely blocked not only NPP production but 10-deoxyNPP biosynthesis, indicating that the loss of N-acetylglucosamine has an impact on the downstream post-PKS modification process. When the ESK631 (*ΔnppYΔnppL*) was complemented by introduction of the *nppY* gene, the 10-deoxyNPP production was restored, suggesting that the hydroxylation at the C10 position was performed as the final step of NPP biosynthesis after the completion of two glycosylation reactions. This observation was also proved through the expression of the *nppDI* gene in the ESK621 (*ΔnppDIΔnppL*) mutant, leading to the production of 10-deoxyNPP (S4 Fig).

Recently, *nypY* from *Pseudonocardia* sp. P1 was heterologously expressed in various strains of *S*. *nodosus* allowed conversion of amphotericin A, B and 7-oxo-amphotericin B to disaccharide-modified forms, yet the conversion yield of these polyene analogues were extremely low [28]. Similarly, we also attempted to introduce *nppY* to generate the disaccharide forms of nystatin analogues in *S*. *noursei*. However, the undetectable levels of nystatin-like polyene derivatives were observed, even though the *nppY* gene was cloned into pSET152 vector under the control of the constitutive promoter, ermE* promoter (S5 Fig). Based on the NPP post-PKS tailoring steps that we propose here, we could hypothesize that NppY recognizes 10-deoxynystatin as a preferred substrate than nystatin itself in *S*. *noursei*.

To deduce the critical regions of NppY, various site-directed mutagenesis analysis were pursued. In general, GT reveals high structure similarity and are classified as a GT-A or GT-B fold. Interestingly, most of the GTs involved in natural product biosynthesis is known to employ a GT-B fold [34]. These proteins are composed of N-terminal domain responsible for the acceptor binding and the C-terminal domain for the sugar donor binding [16, 35, 36]. Through site-directed mutagenesis, arginine at the position of 200 and seven consecutive amino acids in N-terminal were proposed to play significant roles in NppY enzyme activity. In addition, the fusion protein containing the N-terminal half of NppY (presumably involved in 10-deoxynystatin binding) and the C-terminal half of the NppDI (presumably involved in mycosamine transfer) also induced significant decrease of NPP production, implying that the C-terminal domain in NppY might be better suitable for the *N*-acetyl-glucosamine transfer.

Through combination of deletions and complementations of *nppDI*, *nppY*, and *nppL*, the rational post-PKS tailoring pathway of the disaccharide-containing polyene such as NPP has been proposed for the first time. A NPP polyene aglycone is first produced by PKS enzymes, and then sequentially modified by two separate GT enzymes, followed by the regio-specific hydroxylation at the NPP C10 position by the specific P450 hydroxylase. The results shown in this paper not only increased our understanding on polyene post-PKS biosynthetic mechanism, but also provided the second sugar extending GT-driven application for the biosynthesis of novel toxicity-reduced polyene compounds in various actinomycetes.

## Supporting Information

S1 Fig Deletion and complementation of *nppY*. (DOC)

S2 Fig Comparison of amino acid sequence between NppY and TDP-vancosaminyl-transferase GtfD. (DOC)

S3 Fig Deletion and complementation of *nppL*. (DOC)

S4 Fig HPLC analyses of single, double, and triple knockout mutants and their complemented strains. (DOC)

S5 Fig HPLC profiles of the compounds produced by *S*. *noursei* and the *nppY* overexpressed *S*. *noursei* strain. (DOC)

S1 Table Oligonucleotides used for construction of target gene deletion mutants, complementation and introduction of mutagenesis in *nppY* gene. (DOC)

S2 Table Classified glycosyltransferases (GTs) in *P*. *autotrophica* whole genome. (DOC)

S3 Table Basic BLAST result of NppY glycosyltransferase from *P*. *autotrophica*. (DOC)
