# Supplementary material for: Post-PKS Tailoring Steps of a Disaccharide-Containing Polyene NPP in Pseudonocardia autotrophica
Source: PLoS One. 2015 Apr 7;10(4):e0123270. doi: 10.1371/journal.pone.0123270 (PMC4388683; doi:10.1371/journal.pone.0123270)
Supplement: S1 Fig — (DOC) [file pone.0123270.s001.doc]

**S1 Fig.** Deletion and complementation of *nppY*. (A) The absence of *nppY* gene deletion was confirmed by PCR analysis using the check primer F and R; lane 1, 1kb DNA ladder; lane 2, *P. autotrophica* wild-type genomic DNA; lane 3, pDELY; lane 4~6, ESK6011 genomic DNA. (B) HPLC profiles of the compounds produced from ESK6011 and *S. noursei nysL* mutant [29] (left) and an accurate mass spectrum for the polyene derivative from ESK6011 (right). (C) Genetic confirmation of complementation with pPY in ESK6011 mutant using PCR; lane 1, 1kb DNA ladder; lane 2, ESK6011 genomic DNA; lane 3, pPY; lane 4 and 5, ESK6012 genomic DNA.

**
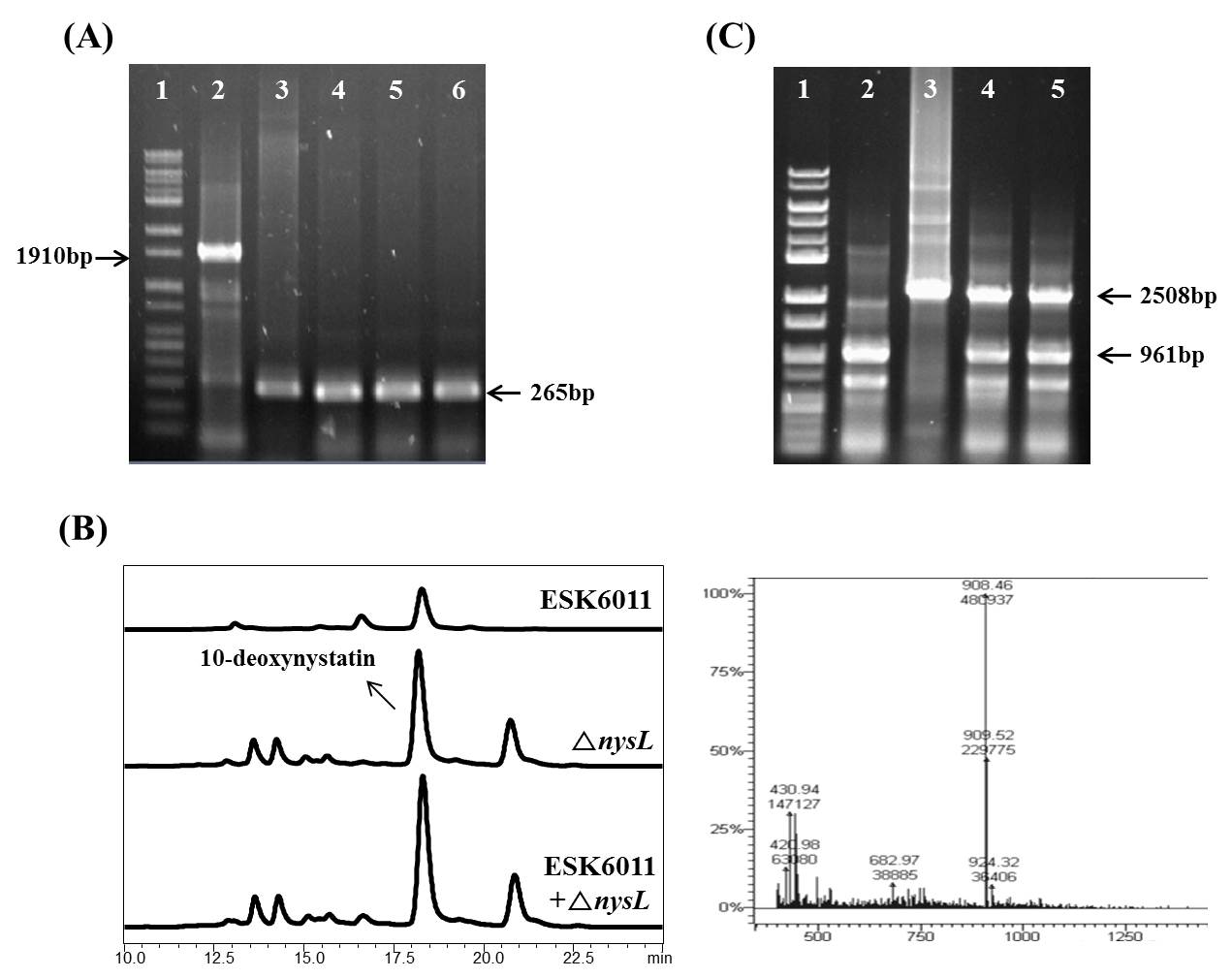
**
