# Supplementary material for: Post-PKS Tailoring Steps of a Disaccharide-Containing Polyene NPP in Pseudonocardia autotrophica
Source: PLoS One. 2015 Apr 7;10(4):e0123270. doi: 10.1371/journal.pone.0123270 (PMC4388683; doi:10.1371/journal.pone.0123270)
Supplement: S2 Fig — (DOC) [file pone.0123270.s002.doc]

**S2 Fig.** Comparison of amino acid sequence between NppY and TDP-vancosaminyl-transferase GtfD. Conserved (asterisk) and homologous (colon) amino acid are marked. Critical regions of GtfD protein (gray highlight) included active sites, acceptor binding sites, and activated NDP-sugar donor binding sites.

**
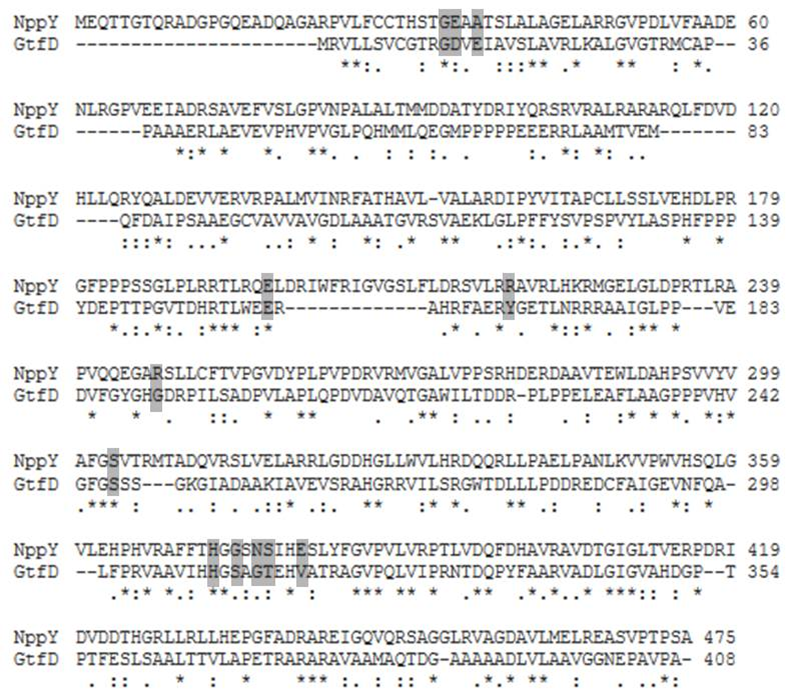
**
