# Supplementary material for: Post-PKS Tailoring Steps of a Disaccharide-Containing Polyene NPP in Pseudonocardia autotrophica
Source: PLoS One. 2015 Apr 7;10(4):e0123270. doi: 10.1371/journal.pone.0123270 (PMC4388683; doi:10.1371/journal.pone.0123270)
Supplement: S3 Fig — (DOC) [file pone.0123270.s003.doc]

**S3 Fig.** Deletion and complementation of *nppL*. (A) The absence of *nppL* gene deletion was confirmed by PCR analysis; lane 1, *P. autotrophica* wild-type genomic DNA; lane 2, pDELL; lane 3, ESK6021 genomic DNA. Genetic confirmation of complementation in ESK6021 was also performed by PCR with pSET152 check primer pairs; lane 4, ESK6021 genomic DNA; lane 5, pPL; lane 6 and 7, ESK6022 genomic DNA. (B) HPLC profiles of compounds isolated from ESK6021 and ESK6022. (C) HPLC-MS analysis and chemical structure with expected MW of 10-deoxynystatin production in ESK6021.

**
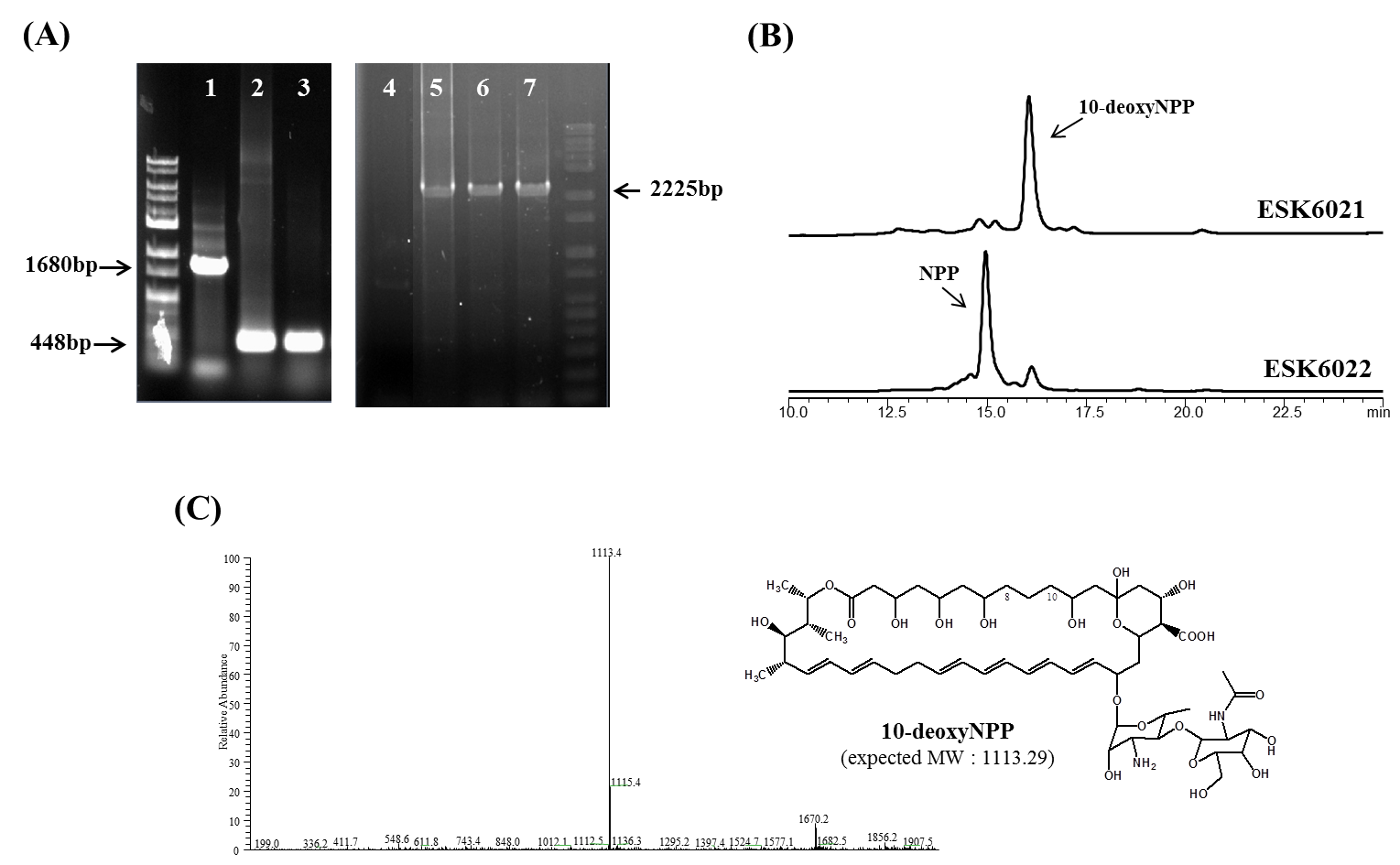
**
