# Supplementary material for: Post-PKS Tailoring Steps of a Disaccharide-Containing Polyene NPP in Pseudonocardia autotrophica
Source: PLoS One. 2015 Apr 7;10(4):e0123270. doi: 10.1371/journal.pone.0123270 (PMC4388683; doi:10.1371/journal.pone.0123270)
Supplement: S5 Fig — (DOC) [file pone.0123270.s005.doc]

**S5 Fig**. HPLC profiles of the compounds produced by *S. noursei* and the *nppY* overexpressed *S. noursei* strain.

**
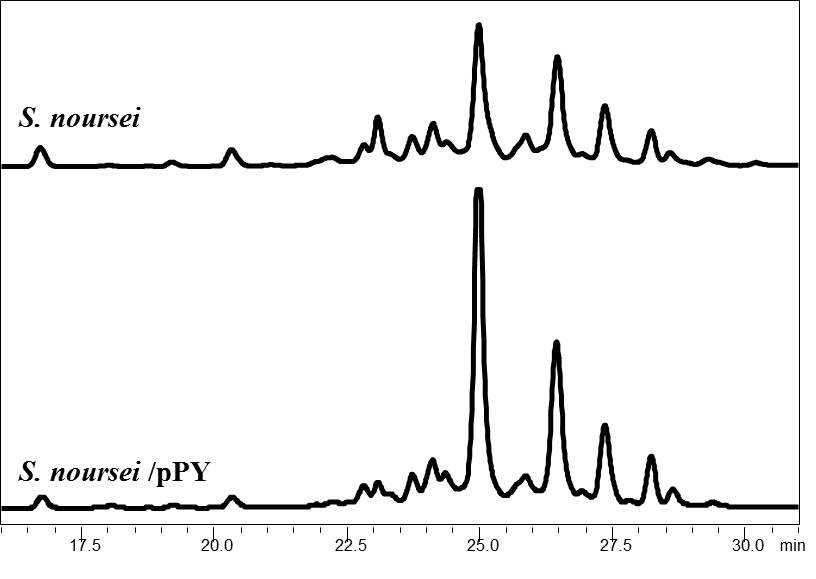
**
