# Supplementary material for: Post-PKS Tailoring Steps of a Disaccharide-Containing Polyene NPP in Pseudonocardia autotrophica
Source: PLoS One. 2015 Apr 7;10(4):e0123270. doi: 10.1371/journal.pone.0123270 (PMC4388683; doi:10.1371/journal.pone.0123270)
Supplement: S1 Table — (DOC) [file pone.0123270.s006.doc]

**S1 Table. Oligonucleotides used for construction of target gene deletion mutants, complementation and introduction of mutagenesis in *nppY* gene.**

| **Purpose** | **Oligonucleotide** | **Sequence^a^** |
| --- | --- | --- |
| Gene deletion | DELY_1F | 5’-GAATTCCGAGGACTGGGACATCGC-3’ |
|  | DELY_1R | 5’-GGATCCCGCCGTGCTGATGGAGCT-3’ |
|  | DELY_2F | 5’-GGATCCAGTGGGTACAGCAGAACAGG-3’ |
|  | DELY_2R | 5’-AAGCTTAACCACGACCTGTGGACCCA-3’ |
|  | DELY CHECK_1F | 5’-TGCTGAACTTCCTGCCGCTG-3’ |
|  | DELY CHECK_1R | 5’-CTTCGACGAGGCCGAGATCG-3’ |
|  | DELY CHECK_2F | 5’-TGGACGTGGAGCATGCAGCT-3’ |
|  | DELY CHECK_2R | 5’-CCAGCTTCGTGATCGCGCAC-3’ |
|  | DELL_1F | 5’-GAATTCCGTCCTGTACTCGTCGGT-3’ |
|  | DELL_1R | 5’-CTGCAGTCATGACGCGTCCTCCGT-3’ |
|  | DELL_2F | 5’-CTGCAGACGCGGTCACGATGGCGC-3’ |
|  | DELL_2R | 5’-AAGCTTACCTGGCCGAGCAGATGG-3’ |
|  | DELL CHECK_1F | 5’-CTGTGCGAGGAGCTGACCGA-3’ |
|  | DELL CHECK_1R | 5’-ACCTGGTGCGGCTGGAGATG-3’ |
|  | DELL CHECK_2F | 5’-CTCAACAGCGCGCGGATGTC-3’ |
|  | DELL CHECK_2R | 5’-CGCGCACCAGGGTGATCATG-3’ |
| Complem-entation | PY_F | 5’-CGGGATCCCGACCGGGGCCTGCTCGTCA-3’ |
|  | PY_R | 5’-GCTCTAGAGCCGGGTGGTCCCGCTGGTGG-3’ |
|  | PL_F | 5’-TCTAGACGACGCGATCGAGACC-3’ |
|  | PL_R | 5’-TCTAGAATCGTGACCGCGTGCC-3’ |
|  | PDIY_1R | 5’-GGCCCCGGTCGGGATCCGACTCCCTGGTTCGGTGG-3’ |
|  | PDIL_2F | 5’-ACCAGGGAGTCGGATCCTCTAGACGACGCGATCGAGACCTG-3’ |
| NppY modifica-tion | YM1_1R | 5’-AACCCGCGCGGCGTGTAGGACCTCGCGAACGGCAGGTCGTGCTCG  ACGAGGCTGCTGAGCA-3’ |
|  | YM1_2F | 5’-ACCTGCCGTTCGCGAGGTCCTACACGCCGCGCGGGTTCCCCCCGC  CGTCGTCCGGGCTGCC-3’ |
|  | YM2_F | 5’-ACCAGTCTCGCGCTCGCC-3’ |
|  | YM2_R | 5’-GTTGGCCTCCCCGGTGGA-3’ |
|  | YM3_F | 5’-AAGCTGGACCGGATCTGGTTC-3’ |
|  | YM3_R | 5’-CTGGCGCAGGGTGCGGCG-3’ |
|  | YM4_F | 5’-AACATCTGGTTCCGGATCGGCG-3’ |
|  | YM4_R | 5’-GTCCAGCTCCTGGCGCAG-3’ |
|  | YM5_F | 5’-TGGTTCCGGATCGGCGTG-3’ |
|  | YM5_R | 5’-CTGCCGGTCCAGCTCCTG-5’ |
|  | YM6_F | 5’-GCCGTCCGGCTGCACAAG-3’ |
|  | YM6_R | 5’-GACGCGCAGCACCGAACGGTC-3’ |
|  | YM7_F | 5’-GAGTCCCTGCTCTGCTTCACCG-3’ |
|  | YM7_R | 5’-GGCGCCCTCCTGCTGGAC-3’ |
|  | YM8_1R | 5’-CCCCGAAGTAGAGGCTCTCGTG**GTA**GCTGTTGCTCCCCCCGTGGG  TGAAGAACGCCCGGA-3’ |
|  | YM8_2F | 5’-AGCAACAGCTACCACGAGAGCCTCTACTTCGGGGTT-3’ |
|  | YM9_1R | 5’-GAGCGTCCGGATCCGGAACCAGATCCGGTCCAG-3’ |
|  | YM9_2F | 5’-CGGATCCGGACGCTCGGC-3’ |
| ^a^Restriction enzyme sites are underlined. | | |
