# Supplementary material for: Post-PKS Tailoring Steps of a Disaccharide-Containing Polyene NPP in Pseudonocardia autotrophica
Source: PLoS One. 2015 Apr 7;10(4):e0123270. doi: 10.1371/journal.pone.0123270 (PMC4388683; doi:10.1371/journal.pone.0123270)
Supplement: S2 Table — (DOC) [file pone.0123270.s007.doc]

**S2 Table. Classified glycosyltransferases (GTs) in *P. autotrophica* whole genome.**

| **Function** | **Count** |
| --- | --- |
| NppDI | 1 |
| Macrolide glycosyltransferase | 1 |
| N-acetylglucosaminyltransferase | 3 |
| UDP-glucuronosyltransferase | 5 |
| Related cell division | 55 |
| Etc. | 47 |
| Function of NppDI was previously confirmed (Lee *et al.*, 2012). | |
