# Supplementary material for: Post-PKS Tailoring Steps of a Disaccharide-Containing Polyene NPP in Pseudonocardia autotrophica
Source: PLoS One. 2015 Apr 7;10(4):e0123270. doi: 10.1371/journal.pone.0123270 (PMC4388683; doi:10.1371/journal.pone.0123270)
Supplement: S3 Table — (DOC) [file pone.0123270.s008.doc]

**S3 Table. Basic BLAST result of NppY glycosyltransferase from *P. autotrophica.***

| **Protein** | **Strain** | **Identity**  (%) | **Similarity** (%) | **E-value** |
| --- | --- | --- | --- | --- |
| NypY | *Pseudonocardia* sp. P1 | 83 | 88 | 0.0 |
| PegA | *Couchioplanes caeruleus* | 51 | 64 | 7e-134 |
| NppDI | *Pseudonocardia autotrophica* | 42 | 57 | 1e-114 |
| * Based on NCBI protein blast search (http://blast.ncbi.nlm.nih.gov) | | | | |
